# NCL Disorders: Frequent Causes of Childhood Dementia

**Published:** 2013

**Authors:** Angela SCHULZ, Alfried KOHLSCHÜTTER

**Affiliations:** 1 Professor of Pediatrics Department of Pediatrics, University Medical Center Hamburg-Eppendorf, Hamburg, Germany

**Keywords:** NCL, Causes, Dementia, Childhood

## Abstract

Dementia in children or young adults is most frequently caused by neuronal ceroidlipofuscinoses (NCL), a group of incurable lysosomal storage disorders linked by the accumulation of a characteristic intracellular storage material and progressive clinical deterioration, usually in combination with visual loss, epilepsy, and motor decline. The clinical characteristics can vary and the age at disease onset ranges from birth to over 30 years. Diagnosis of an NCL is difficult because of genetic heterogeneity with14 NCL forms (CLN1-CLN14) identified and a high phenotype variability. A new classification of the disorders is based on the affected gene and the age at disease onset and allows a precise and practicable delineation of every NCL disease. We present a clear diagnostic algorithm to identify each NCL form. A precise diagnosis is essential for genetic counseling of affected families and for optimizing palliative care. As patient management profits from recognizing characteristic complications, care supported by a specialized team of NCL clinicians is recommended.

The development of curative therapies remains difficult as the underlying pathophysiological mechanism remains unclear for all NCL forms.

## Introduction

Unexpected loss of cognitive abilities (dementia) in young patients presents a diagnostic challenge. It is most frequently caused by neuronal ceroidlipofuscinoses (NCL), a group of lysosomal storage disorders linked by the accumulation of a characteristic storage material in neurons and other cell types, and a progressive clinical deterioration, usually in combination with visual loss, epilepsy, and motor decline (1). Diagnosis of an NCL is difficult because of a high number of different NCL-causing genes and significant genotype phenotype variability. Nevertheless, the specific diagnosis of an NCL disease can be made in a time- and cost-effective manner using very few diagnostic steps.

## New nomenclature of NCL diseases

Traditionally, NCL diseases were classified according to the age at disease onset into congenital, infantile, late infantile, juvenile, and adult forms and sometimes also according to the respective authors (Haltia-Santavuori, Jansky-Bielschowsky, Batten, Spielmeyer-Vogt, Kufs) (2). More recently it became clear that NCL diseases are much more heterogeneous than previously thought and that mutations in the same gene can lead to very different clinical courses (3, 4). Designations such as “Finish” or “Turkish” NCL variants are also not useful any longer, as mutations in the respective genes have been shown to occur worldwide (5).The traditional nomenclature has therefore become obsolete and has recently been replaced by an internationally developed combined genetic and clinical classification of NCL disorders (table 1) (2). It classifies both the defective gene as well as the age at disease onset (congenital, infantile, late infantile, juvenile or adult). An exact diagnosis is essential for genetic counseling, sharing information regarding prognosis and optimal palliative care.

**Table 1:** Classification of NCL Disorders According to Genetic and Clinical Characteristics

**Disease**	**MIM** **Number / reference**	**Gene designation**
CLN1 disease, infantile CLN1 disease, late-nfantile CLN1 disease, juvenile CLN1 disease, adult	256730	CLN1 (PPT1)
CLN2 disease, late-infantile CLN2 disease, juvenile	204500	CLN2 (TPP1)
CLN3 disease, juvenile	204200	CLN3
CLN4 disease, adult (autosomal dominant)	162350	CLN4 (DNAJC5)
CLN5 disease, late-infantile CLN5 disease, juvenile CLN5 disease, adult	256731	CLN5
CLN6 disease, late-infantile CLN6 disease, adult (Kufs type A)	601780	CLN6
CLN7 disease, infantile	610951	CLN7 (MFSD8)
CLN8 disease, late-infantile CLN8 disease, EPMR	600143	CLN8
CLN10 disease, congenital CLN10 disease, late- infantile CLN10 disease, juvenile CLN10 disease, adult	610127	CLN10 (CTSD)
CLN11 disease, adult	138945	CLN11 (GRN)
CLN12 disease, juvenile	(9)	CLN12 (ATP13A2)
CLN13 disease, adult (Kufs type B)	(8)	CLN13 (CTSF)
CLN14 disease, infantile	(11)	CLN14 (KCTD7)

## The genetic spectrum of NCL diseases

To date, 14 different NCL forms have been To date, 14 different NCL forms have been described (table1) (5-10). More NCL genes remain to be identified as in some patients mutations cannot be demonstrated in any of the known NCL genes although they present with typical NCL symptoms and characteristic cellular storage material. Intracellular localization and- if known- function of the defective NCL proteins are different: Four NCL types are caused by defects in lysosomal enzymes (CLN1, CLN2, CLN10, CLN13), others by defects in transmembrane proteins (CLN3, CLN6, CLN7, CLN8) (6). Also, mutations of an ATPase (CLN12) (9) or a potassium channel (CLN14) (11) seem to cause NCL disease. The CLN4 gene (also called DNAJC5) codes for a protein with putative function in synapses (10). The exact mechanism, how these defects lead to neurodegeneration, is still not understood. Despite their genetic heterogeneity, the different NCL diseases have clinically much in common. This is important both for diagnosis and palliative treatment. Curative therapies do not yet exist for any NCL disease.

## The clinical spectrum of NCL diseases

In almost all NCL forms the patients initially present healthy and have a normal development. Main symptoms are a combination of dementia, visual loss, epilepsy, and motor deterioration that progress slowly but inexorably. The age at disease onset can range from birth till young adulthood. The order in which symptoms occur is variable. In a young child, first symptoms are standstill and regression of psychomotor development, or intractable epilepsy. In a school child, first symptoms are mostly visual loss, followed by dementia. The different disease courses are described below (2).For diagnostic tests refer to table 2. 

**Table 2:** Diagnostic Algorithm for NCL Diseases

Clinical presentation	Necessary diagnostic tests	Possibly affected genes
**Newborn** With epilepsy and microcephaly	Enzyme testing for cathepsin D (CtsD) (leucocytes fibroblasts)	CtsD deficient: CLN10
**Young child** (>6months) with developmental or regression and/or newly severe epilepsy of unknown cause	Enzyme testing for PPT1 and TPP1 (dry blood spots; confirmation in leucocytes or fibroblasts)	
	PPT1	PPT1 deficient: CLN1
	TPP1	TPP1 deficient: CLN2
	If PPT1 and TPP1 enzyme normal: Electron microscopic examination (skin biopsy or lymphocytes): If storage material is present: genetic testing.	CLN5, CLN6, CLN7, CLN8, CLN14 (KCTD7)
**School child** with visual loss and / or dementia and epilepsy	Search for lymphocyte vacuoles (light microscopy of blood smear). If lymphocyte vacuoles are present: genetic testing of the CLN3 gene	CLN3
	If no lymphocyte vacuoles, enzyme testing for PPT1, TPP1 and CtsD (see above)	PPT1 deficient: CLN1, TPP1 deficient: CLN2, CtsD deficient: CLN10
	If PPT1 and TPP1 enzyme normal: Electron microscopic examination (skin biopsy or lymphocytes). If storage material is present: genetic testing.	CLN5, CLN6, CLN7, CLN8, CLN12 (ATP13A2)
**Young adult** with non-specific mental, motor or behavioral abnormalities.	Enzyme testing for PPT1, TPP1 and CtsD (see above)	PPT1 deficient: CLN1, TPP1 deficient: CLN2, CtsD deficient: CLN10
	If PPT1 and TPP1 enzyme activity is normal: Electron microscopic examination (skin biopsy or lymphocytes). If storage material is present: genetic testing (eventually in special cases even without detection of storage material), consider possible mode of inheritance.	If autosomal dominant:CLN4 (DNAJC5) If autosomal recessive: CLN6, CLN11 (GRN, CLN13 (CTSF)

**Congenital disease onset**

This is the only NCL form where patients are severely affected already at birth. Intrauterine or immediate postnatal onset of epileptic seizures as well congenital microcephaly should lead to the suspected diagnosis. 

Confirmation is based on demonstration of cathepsin D deficiency and a mutation in the CLN10 gene. The disease leads to death in early infancy. 

**Infantile disease onset**

First symptoms are standstill and subsequent regression of psychomotor development occurring at the age of 10-18 months. These are followed by muscular hypotonia which may change to spasticity later on, as well as myoclonus and epilepsy. Fulminant brain atrophy leads to progressive microcephaly. The electroencephalogram (EEG) becomes flat. Visual contact is lost due to retinopathy. Diagnosis depends on demonstration of a lysosomal enzyme defect (PPT1) and a CLN1 mutation (see table 2). Death occurs in early childhood. 

**Late infantile disease onset**

**Fig 1:**
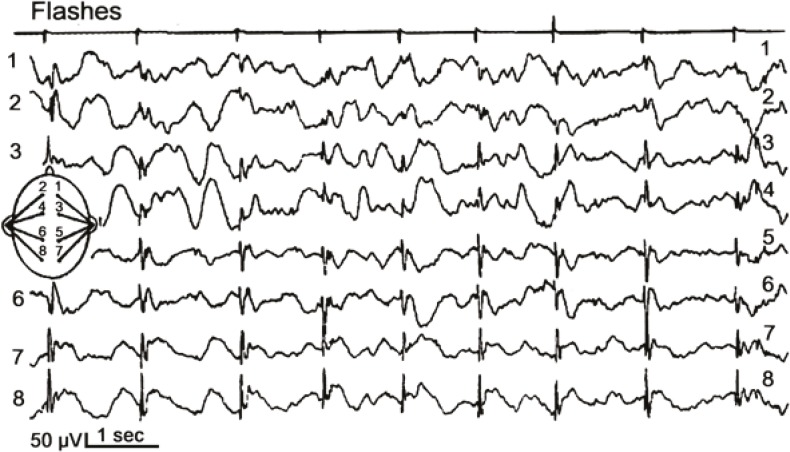
Electroencephalogramm of a 5-year-old patient with CLN2 disease, late infantile, recorded under slow (1/sec) photic stimulation. Spikes over the occipital areas correspond to light flashes and illustrate increased neuronal excitability during this stage of the disease

**Fig 2:**
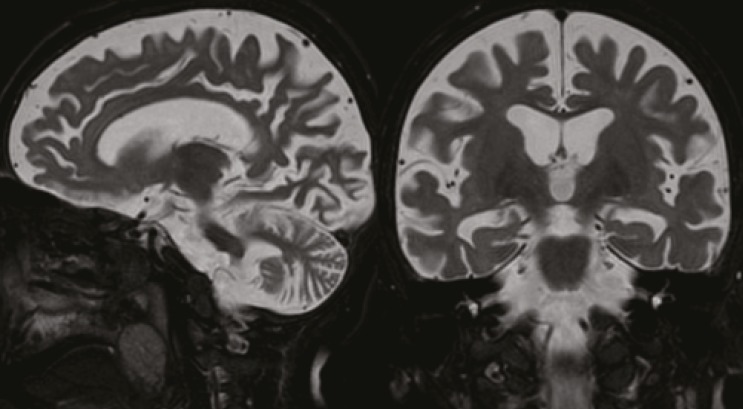
Magnetic resonance tomograms, T2-weighted, of a 5½-year-old child with CLN2 disease, late infantile. Cerebral and cerebellar atrophy is evident

In this age group, several different gene defects and enzyme deficiencies may be responsible (table 2). First symptoms such as muscular hypotonia, ataxia and developmental regression occur at the age of 2-3 years. At the same age, therapy-resistant epileptic seizures frequently appear. The EEG may show posterior spikes under slow photic stimulation in several NCL forms (CLN2, CLN5, CLN6) (Fig. 1). Cerebral and cerebellar atrophy are seen by cerebral magnetic resonance imaging (Fig. 2). Visual loss is often diagnosed with delay due to the severe neurologic deficits of the patients. Additional problems are increasing spasticity and non-epileptic myoclonus. Most patients live until the age of 10-15 years.

**Juvenile disease onset**

**Fig 3:**
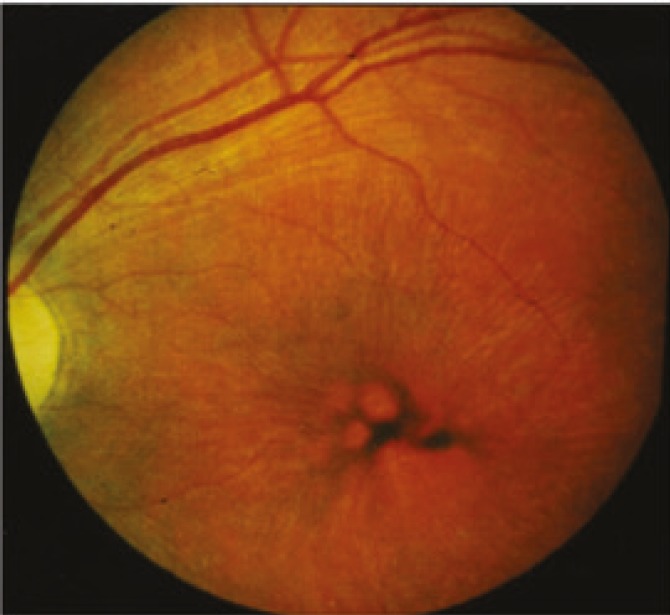
Fundoscopic appearance of the retina of a patient with CLN3 disease, juvenile. Irregular pigment distribution and thin blood vessels are visible

**Fig 4:**
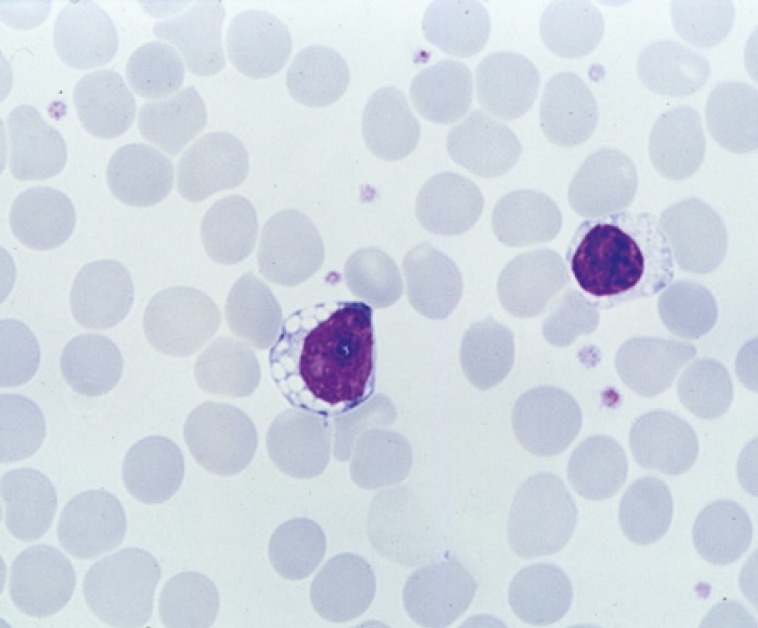
Vacuoles in the cytoplasm of a peripheral blood lymphocyte from a patient with CLN3 disease, juvenile, routine blood smear

The juvenile disease typically starts with isolated visual failure due to retinal degeneration at early school age (5-7 years). An early ophthalmologic finding is the extinguished electroretinogram, later also a pigmentary retinopathy with thinning of vessels (frequently mistaken for “retinitis pigmentosa”, Fig. 3). Most patients in this group have an underlying CLN3 mutation and vacuolated lymphocytes in their peripheral blood (Fig. 4). Unexpected difficulties with abstract reasoning at school herald progressive dementia, grand mal seizures and motor difficulties appear, the latter mostly of a parkinsonian type with small-stepped gait and rigor. Spasticity and myoclonus are rare but may develop late during the disease course. During puberty a psychoorganic syndrome may occur with attacks of fear, panic, and hallucinations or with depressive or aggressive mood changes. These disturbances are a particular challenge to psychiatric management. Older patients may suffer from cool, livid extremities due to sinus bradycardia or other circulatory disturbances. Improved palliative management (e.g., gastric feeding tube for swallowing difficulties) has increased life expectancy up to the age of 30 years and more. 

**Adult disease onset**

The age at disease onset in adult NCL forms has a wide range with average around 39 years (12, 13). First symptoms are variable. Clinically, a type A (initial progressive myoclonus epilepsy, dementia and ataxia) and type B (initial behavioral abnormalities and motor problems) have been differentiated, both without visual problems (12). The identification of affected genes as well as the number of diagnosed cases is currently increasing.

## Special aspects of palliative therapy in NCL

Palliative management of a patient with an NCL disease represents a significant challenge due to a number of clinical problems some of which are disease-specific. Moreover, recognition of some complications is difficult as most patients are not able anymore to communicate verbally at later stages of their disease. Episodes of apparent pain require thorough clinical examination. Abdominal pain may be caused by constipation due to disease-related intestinal hypomotility or inadequate nutrition, but many other causes must be considered, such as unnoticed bone fractures or renal calculi, which both are a frequent consequence of immobility. Attacks of exacerbated spasticity (“spastic crisis”) may caused by gastro-esophageal reflux or an acute event such as testicular torsion. Collaboration with other physicians experienced in clinical NCL problems can help improving palliative medication. Table 3, which is based on the authors experience, gives an overview on palliative medication for treating the most common symptoms in NCL.

**Epilepsy**

Seizures in NCL are mostly treatment-resistant. Some commonly used antiepileptic drugs may have unusual effects on a patient whose brain is undergoing a degenerative process. We suggest the following guidelines for treating seizures in NCL: 

(1) Complete absence of seizures or normalization of electroencephalogram are not realistic aims in NCL patients.

(2) The EEG in NCL serves mainly for monitoring. Therapy should be adjusted to clinical symptoms. 

(3) Therapy with more than two antiepileptic drugs may lead to irritating side effects rather than to reduction of seizures.

(4) Some antiepileptic drugs are recommended in NCL patients (valproate and lamotrigine, table 3), others may have negative effects on the disease course and should be avoided (carbamazepin, phenytoine, vigabatrin).

(5) “Use only as many drugs as are necessary and as few as possible.”

**Myoclonus**

Myoclonus is a symptom which is difficult to treat. Patients, however, frequently seem to be less disturbed by the myoclonic jerks than caregivers. Levetiracetame, zonisamide and piracetame are effective treatments (table 3). Benzodiazepins should be avoided. Some antiepileptic drugs can aggravate myoclonus. Therefore, reduction of the number of different medications may improve myoclonus.

**Spasticity**

Painful spasticity is another symptom which should be treated: Substances such as baclofen and are effective. Tetrahydrocannabinol may be used as add on medication (table 3). Benzodiazepin as well as tetrazepam seem to be less effective (less potency, negative effect on disease course, increased mucous secretion). Physical therapies and the search for any underlying trigger of spastic crises should always be considered.

**Table 3:** Overview on Medication for Palliative Therapies in NCL

**Symptom**	**Substance**	**Comment**
Epilepsy*	Valproate	Advantage: mood stabilizing effect, useful in juvenile NCL patients with psychotic symptoms
	Lamotrigine	
	Topiramate	Increase dosage SLOWLY to minimize side effects such as speech disturbance (starting dose 0.5 mg/kg/d). Agitation may be a side effect. In this case discontinue the drug.
	Levetiracetame	Severe agitation is a possible side effect in juvenile NCL.
	Diazepam, lorazepam	Acute therapy of prolonged grand mal seizures
Myoclonus	Levetiracetame	Also effective as anti-epileptic medication (especially in late infantile NCL)
	Zonisamide	
	Piracetame	High dosage required (300-350 mg/kg/d)
Spasticity	Baclofen (1st choice)	Frequently high dosagerequired
	Tizanidine (2nd choice)	Good effect also against dyskinesia
	Tetrahydrocannabinol	“Add-on” medication, increase dosage SLOWLY up to 0.07 mg/kg/d,
	Botulinumtoxine	Local application by injection to muscles; always accompanied by physical therapy

* Avoid overtreatment, as most seizures in NCL are treatment-resistant

**Other problems**

Diagnosis and treatment of tormenting psychopathological symptoms such as sleep disturbances, fear, aggressive behaviour, depression, and hallucinations represent special challenges. Psychopharmacologic medication should be given in collaboration with child neurologists and child psychiatrists, parents, and caregivers. Only this way, side effects of drugs as well as undetected causes for fear and restlessness can be identified in time.
